# Molecular Diagnostics Supporting a ≥35% Diffuse Peritubular Capillaritis Extent Threshold for Diagnosis of AMR—A Retrospective Dual Center Study

**DOI:** 10.3390/ijms262210945

**Published:** 2025-11-12

**Authors:** Michael Eder, Marian C. Clahsen-van Groningen, Michael Mengel, Haris Omic, Daniel Cejka, Benjamin Adam, Nicolas Kozakowski, Željko Kikić

**Affiliations:** 1 Division of Nephrology and Dialysis, Department of Internal Medicine III, Medical University of Vienna, Währinger Gürtel 18–20, 1090 Vienna, Austria; 2 University Hospital RWTH Aachen, Department of Nephrology, 52074 Aachen, Germany; 3 Department of Pathology and Clinical Bioinformatics, Erasmus MC Transplant Institute, 3015 GD Rotterdam, The Netherlands; 4 Laboratory for Pathology (PAL), 3318 AL Dordrecht, The Netherlands; 5 Department of Laboratory Medicine and Pathology, University of Alberta, Edmonton, AB T6G 2R3, Canada; 6 Department of Medicine III, Nephrology, Hypertension, Transplantation and Rheumatology, Ordensklinikum Linz-Elisabethinen, 4010 Linz, Austria; 7 Department of Pathology, Medical University of Vienna, 1090 Vienna, Austria; 8 Department of Urology, Medical University of Vienna, 1090 Vienna, Austria

**Keywords:** nephrology, kidney transplantation, antibody-mediated rejection, intragraft gene expression, peritubular capillaritis

## Abstract

Peritubular capillaritis (ptc) is a hallmark lesion of antibody-mediated rejection (AMR), but the grading of its extent is historically based on arbitrary defined cut-offs. Molecular AMR diagnosis via intragraft gene expression measurements may provide evidence to challenge established ptc categories. We retrospectively included 38 renal allograft biopsies from clinical routine, performed because of suspicion of AMR. Biopsies were re-assessed by an experienced nephropathologist and intragraft gene expression was measured using the NanoString nCounter^®^ platform. Ptc categories were correlated with AMR gene expression to identify a ptc extent cut-off with optimal prediction of molecular diagnosis of AMR [gene expression levels above first quartile (AMR^Q>1^)]. Finally, an independent validation cohort (*n* = 25, Erasmus MC, Rotterdam, The Netherlands) was included to reproduce the results. Re-assessment of biopsies revealed AMR in 26/68.4%, mixed rejection in 5/13.2%, and T-cell-mediated rejection in 3/7.9%. Biopsies with diffuse ptc had significantly higher AMR gene expression compared to biopsies with focal ptc and biopsies with no ptc (64.0/53.3–84.0 vs. 31.5/27.0–49.5, *p* = 0.023 and 27.0/14.3–31.8, *p* = 0.003, median/IQR). Sensitivity analysis revealed that a ≥35% ptc cut-off resulted in higher AUCs for predicting AMR^Q>1^ compared to ptc^50%^ (AUC 0.78, 95% CI: 0.63–0.93, *p* = 0.009 versus AUC: 0.74, CI: 0.56–0.90, *p* = 0.03). In the validation cohort, only the ptc^35–^, but not the ptc^50%^, cut-off significantly predicted AMR^Q>1^ (AUC 0.75, 95% CI: 0.54–0.96 *p* = 0.04 vs. AUC 0.69, CI: 0.46–0.93, *p* = 0.13). Using intragraft gene expression measurement, we identified a new ptc extent threshold with better prediction of molecular AMR. The newly proposed cut-off of ≥35% could potentially improve diagnostic evaluation and prognostication in cases with suspected or diagnosed AMR.

## 1. Introduction

Antibody-mediated rejection (AMR) is an important contributor to late allograft loss [1,2,3]. The histological hallmark lesions of AMR are microvascular inflammation (MVI), including peritubular capillaritis (ptc) and glomerulitis (g). The diagnostic thresholds for ptc are based on the number of inflammatory cells (ptc score) and a minimum 10% percentage of involved peritubular capillaries per biopsy specimen [4]. The interpretation of ptc can be challenging due to limited specificity, as it can also be observed in T-cell mediated rejection (TCMR) [5] or other entities such as BKPyV-associated nephropathy (BKPyVAN) [6]. Although it is recommended to document both the ptc score and the ptc extent, most of the literature has focused on the application of the ptc score alone. However, earlier studies showed that diffuse ptc (extent of inflammation > 50%) was associated with worse graft survival independent from the ptc score, and that the additional integration of diffuse ptc patterns in biopsies with low-grade inflammation may identify patients at higher risk for transplant glomerulopathy and graft loss [7,8].

Despite their broad application, current ptc thresholds were arbitrarily defined and may not accurately reflect pathophysiological phenotypes. It may be speculated that distinct ptc extent cut-offs could be applied in different clinical scenarios, including chronic AMR or TCMR. One of the main aims of the Banff peritubular capillaritis working group was to explore adaptations of the diagnostic and prognostic relevance of the current ptc categories [9]. In this context, the use of molecular rejection markers, which already have been implemented for the diagnosis of AMR, was suggested [10,11]. Yet, as discussed at the 2024 Banff meeting in Paris, France, there is still substantial discrepancy between biopsy-based transcriptomic results and histology assessment, warranting future studies to resolve these controversies (http://dx.doi.org/10.2139/ssrn.5291680, accessed on 6 October 2025).

The aim of this work was therefore to compare the diagnostic performance of a new ptc ≥ 35% versus ≥50% cut-off for predicting gene expression profiles indicative of AMR (AMR^Q>1^) and to explore correlations with g and MVI. We speculate that the assessment of AMR specimens using gene expression analysis would allow for the definition of novel thresholds of ptc extent, reflecting molecular AMR phenotypes more accurately than the current one. We applied one of the most studied systems, the NanoString nCounter^®^ platform, allowing reliable measurement of gene expression profiles in formalin-fixed paraffin-embedded biopsy specimens [12,13,14].

## 2. Results

### 2.1. Baseline Characteristics and Biopsy Findings

The exploratory cohort consisted of nineteen renal transplant recipients who underwent allograft biopsy because of graft dysfunction at the local center (first biopsy 4.2/1.1–42.3 months after transplantation, median/IQR). Each patient underwent two allograft biopsies, resulting in 38 biopsy specimens in total (Medical University of Vienna *n* = 22, Ordensklinikum-Elisabethinen Linz *n* = 16). Mean age at transplantation was 46.6 ± 16.0 (mean ± SD) years. Two patients (5.3%) had pre-formed DSAs at the time of transplantation. At the time of biopsy, DSAs were present in 24 (66.7%) cases. Re-assessment of the included biopsies by a single experienced nephropathologist (M.M), blinded to the clinical results, revealed the following diagnoses: AMR in 26 (68.4%) biopsies, mixed rejection in 5 (13.2%), and TCMR in 3 samples (7.9%). Four (10.5%) biopsies were categorized as no rejection.

Peritubular capillaritis was scored as follows: ptc 0 in 12 (31.6%), ptc 1 in 10 (26.3%), ptc 2 in 14 (36.8%), and ptc 3 in 2 (5.3%) biopsies. Semi-quantitative assessment of the ptc extent revealed focal (10–49%) in 20 (52.6%) samples, diffuse (≥50%) in 6 (15.8%), and no ptc (<10%) in 12 (31.6%) cases. Continuous ptc extent evaluation (ptc^%^) revealed a median ptc^%^ of 25% (IQR 0–40%). Ptc^%^ correlated significantly with glomerulitis (Banff g score); R = 0.53, *p* < 0.001, total interstitial inflammation (Banff ti score); R = 0.49, *p* = 0.002, and tubular atrophy (Banff ct score); R = 0.43, *p* = 0.01. Table 1 lists baseline parameters of included biopsies in detail.

### 2.2. Gene Expression Analysis in the Exploratory Cohort

**Figure 1:**
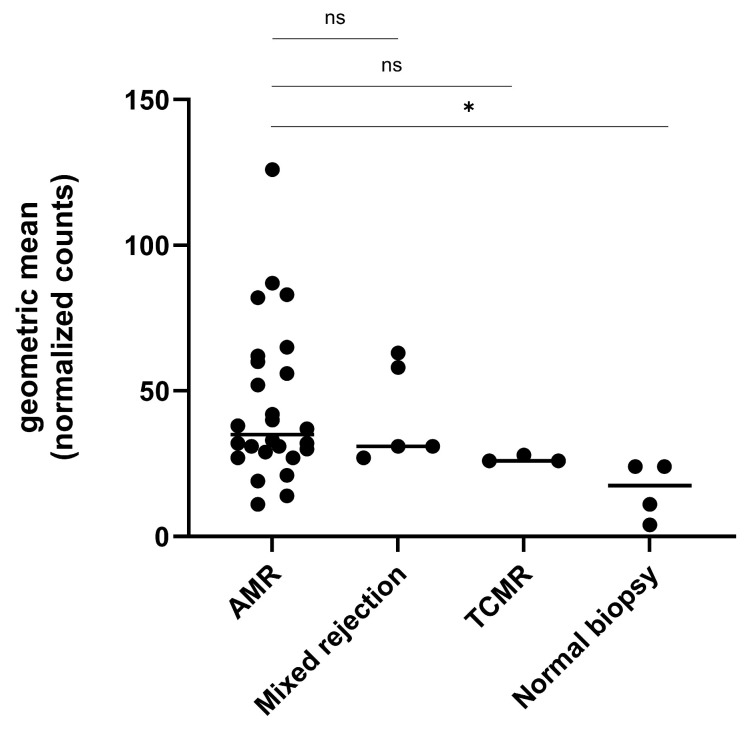
AMR gene expression levels compared between different types of allograft injuries in the exploratory patient cohort. All diagnoses were reassessed by an experienced nephropathologist. Highest gene expression levels were found in biopsies diagnosed with antibody-mediated rejection (AMR), followed by biopsies diagnosed with mixed rejections. AMR gene expression was significantly increased in AMR biopsies compared to normal biopsies, but not significantly higher compared to biopsies with T-cell-mediated (TCMR) or mixed rejections. Abbreviations: AMR: antibody-mediated rejection, TCMR: T-cell-mediated rejection, ns: not significant, and *: *p* < 0.05.

We calculated the AMR gene score for all 38 biopsies. AMR gene expression in relation to histological diagnosis are displayed in Figure 1. AMR gene expression was significantly higher in biopsies diagnosed with AMR (35/28.9–60.5, median/IQR) compared to normal biopsies (17.5/5.8–24, *p* = 0.004), but not significantly higher compared to biopsies with TCMR (26/26–28, *p* = 0.07) or mixed rejections (31/29–60.5, *p* = 0.9). All biopsies were divided into quartiles based on their AMR gene set levels. Two out of three TCMR cases and all biopsies diagnosed as no rejection had AMR gene scores below the first quartile. Subsequently, AMR gene scores above the first quartile (AMR^Q>1^) were considered as a molecular diagnosis of AMR.

### 2.3. Gene Expression Analysis in Relation to Ptc and Other Banff Single Lesions

**Figure 2:**
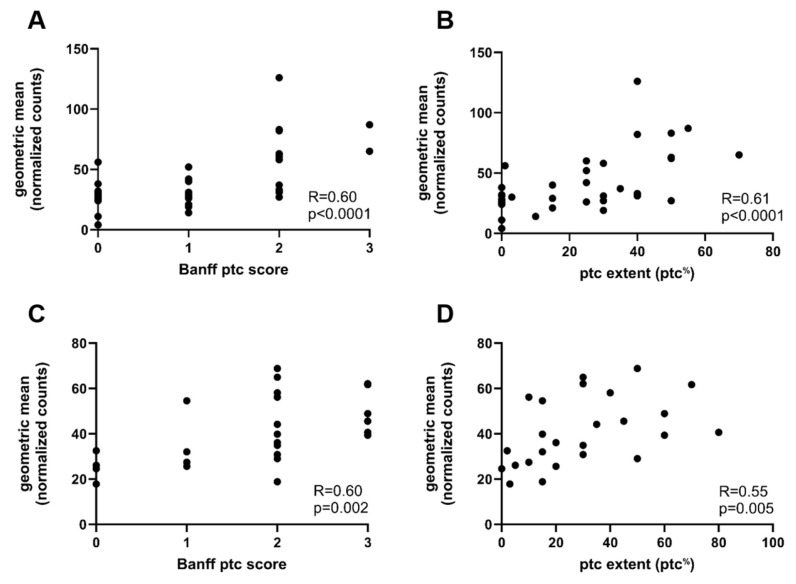
Correlation between AMR gene expression (y-axis), ptc extent (ptc^%^), and the Banff ptc score. (**A**) Spearman correlation between intragraft AMR gene expression levels and the ptc score in the exploratory cohort (x-axis, represents Banff ptc score classification). (**B**) Spearman correlation between AMR gene expression (y-axis) and the Banff extent (x-axis, expressed as continuous parameter, ptc^%^) in the exploratory cohort. (**C**) Spearman correlation between AMR gene expression and the Banff ptc score in the validation cohort (x-axis, represents Banff ptc score classification). (**D**) Spearman correlation between AMR gene expression and the ptc extent (ptc^%^) in the validation cohort (x-axis represents Banff ptc extent expressed as continuous parameter, ptc^%^). Abbreviations: AMR: antibody-mediated rejection, ptc: peritubular capillaritis (Banff lesion score), ptc^%^: peritubular capillaritis extent expressed as continuous parameter, and R = correction coefficient.

**Figure 3:**
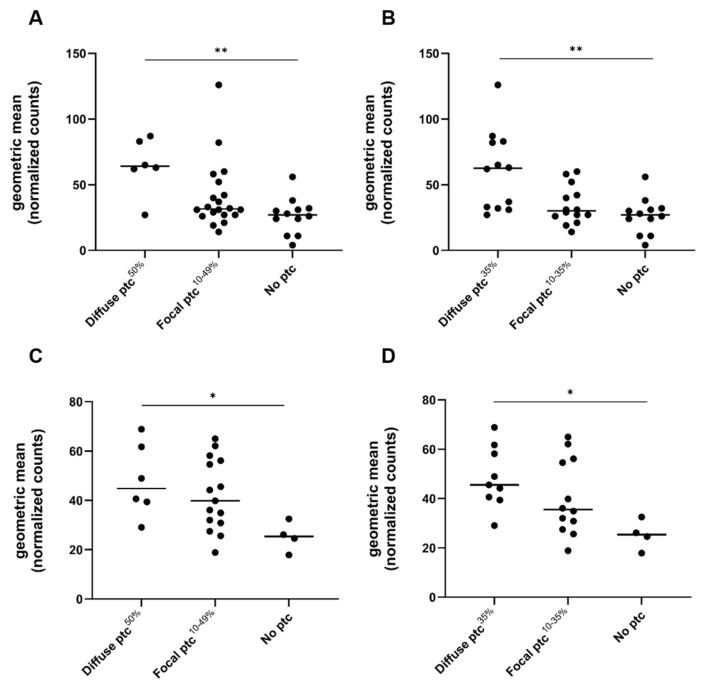
AMR gene expression levels compared between the old (ptc^50%^) and new ptc (ptc^35%^) cut-off. (**A**) AMR gene expression compared between focal and diffuse ptc, applying the established 50% cut-off in the exploratory cohort. (**B**) AMR gene expression compared between focal and diffuse ptc, applying the new ptc 35% cut-off in the exploratory cohort. (**C**) AMR gene expression compared between focal and diffuse ptc, applying the established 50% cut-off in the validation cohort. (**D**) AMR gene expression compared between focal and diffuse ptc, applying the new ptc 35% cut-off. Abbreviations: AMR: antibody-mediated rejection, and ptc: peritubular capillaritis. * indicates *p* < 0.05; ** indicates *p* < 0.01.

In the exploratory cohort, the ptc score correlated significantly with AMR gene expression (R = 0.60, *p* < 0.0001, Figure 2). Biopsies with diffuse ptc had significantly higher AMR gene expression compared to biopsies with focal ptc and biopsies with no ptc (diffuse ptc: 64.0/53.3–84.0 vs. focal ptc: 31.5/27.0–49.5, *p* = 0.023; and no ptc: 27.0/14.3–31.8, *p* = 0.003, median/IQR). Figure 3 illustrates AMR gene expression in relation to the established ptc extent categories (focal 10–49% vs. diffuse ≥ 50%). The continuous ptc (ptc^%^) also correlated significantly with AMR gene expression (R = 0.61, *p* < 0.0001; Figure 2). AMR gene expression also significantly correlated with the Banff g score (R = 0.42; *p* = 0.01) and with the combined microvascular inflammation score (MVI, sum of ptc and g) (R = 0.64, *p* < 0.0001) (Supplemental Figure S1). Other Banff single lesion scores did not correlate with the AMR gene expression (Supplemental Table S3).

### 2.4. ROC Analysis for the Prediction of AMR^Q>1^

**Figure 4:**
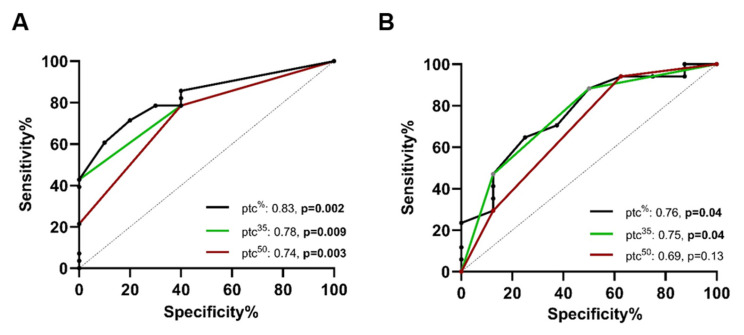
ROC curves for the prediction of AMR gene set levels over the first quartile (AMR^Q>1^) with different ptc cut-offs, as follows: ptc^35%^, ptc^50%^, and ptc extent. Areas under the curve (AUC) were highest with the ptc^%^ in both cohorts. As a threshold, the ptc^35%^ cut-off had higher ROCs compared to the ptc^50%^ in both cohorts. In the validation cohort, only the ptc^%^ and the ptc^35%^ were significantly associated with AMR^Q>1^. (**A**) ROCs analyzed in the exploratory cohort; (**B**) ROCs analyzed in the confirmatory cohort. Bold numbers indicate statistically significant associations. Abbreviations: AMR: antibody-mediated rejection and ptc: peritubular capillaritis.

In the next step, we evaluated the ability of ptc categories to predict molecular AMR diagnosis (AMR^Q>1^). The ptc^%^ as reference resulted in an AUC of 0.83 (95% CI: 0.70–0.96, *p* = 0.002) compared to an AUC of 0.81 (95% CI: 0.67–0.93, *p* = 0.004) for the Banff ptc score, an AUC of 0.81 (95% CI: 0.62–0.995, *p* = 0.004) for the g score, and an AUC of 0.88 (95% CI 0.77–0.99, *p* < 0.001) for the combined MVI score. Sensitivity analysis revealed higher AUCs for the prediction of AMR^Q>1^ when a ptc extent cut-off of ≥35% (ptc^35%^) was used compared to ptc cut-off of ≥50% (ptc^50%^), as follows: ptc^35%^: AUC 0.78, 95% CI: 0.63–0.93, *p* = 0.009, versus ptc^50%^: AUC 0.74, 95% CI: 0.56–0.90, *p* = 0.03; Figure 4 and Table 2. All biopsies with a ptc extent between 35% and 49% had AMR gene set expression levels above the first quartile and did not differ significantly regarding their gene expression levels compared to biopsies with a ptc extent of ≥50% (ptc 35–49%: 35/32–93 versus ptc^50%^: 64/53–84, *p* = 0.59, median/IQR).

### 2.5. Comparison of Biopsies with and Without Transplant Glomerulopathy (cg > 0)

To account for potentially different gene expressions in biopsies with and without transplant glomerulopathy, we performed a secondary analysis including only biopsies with cg > 0 (*n* = 24). Similar to the main analysis, we found a significant correlation between the ptc^%^ and the AMR gene set (R = 0.71; *p* < 0.001). In biopsies with transplant glomerulopathy, the ptc^35%^ cut-off resulted in a numerically but not significantly higher AUC for the prediction of AMR gene expression over the 25th percentile compared with the ptc^50%^ cut-off, as follows: AMR gene set: AUC 0.75 (95% CI: 0.54–0.96) vs. 0.68 (95% CI: 0.43–0.94), *p* = 0.71.

### 2.6. Gene Expression Analysis in the Validation Cohort

To validate our findings, we performed the same analyses in an independent external cohort of 25 biopsies diagnosed with AMR (eight with chronic active AMR), originating from Erasmus MC, Rotterdam. Ptc scores were as follows: ptc 0 in four (16%), ptc 1 in four (16%), ptc 2 in eleven (44%), and ptc 3 in six (24%) samples. Ptc categories were as follows: no ptc in 4 (16%), focal ptc in 15 (60%), and diffuse ptc in 6 (24%) biopsies. In line with the exploratory cohort, both the ptc score and ptc^%^ correlated significantly with AMR gene expression (AMR gene set and ptc score were as follows: R = 0.6, *p* = 0.002, ptc^%^: R = 0.55, *p* = 0.005; Figure 2C,D). AMR gene expression differed significantly between ptc extent categories when applying both the old ptc^50%^ cut-off (diffuse ptc 44.8/36.8–63.5, focal ptc 39.8/30.8–56.1, and no ptc 25.3/19.5–30.9, *p* = 0.036, median/IQR) and the new ptc^35%^ cut-off (diffuse ptc 45.5/40.0–59.9, focal ptc 35.5/28.3–55.8, and no ptc 25.3/19.5–30.9, *p* = 0.02 median/IQR; Figure 3C,D).

### 2.7. ROC Analysis for the Prediction of AMR^Q>1^ in the Validation Cohort

Confirming the findings from the exploratory study, the new ptc cut-off of 35% showed higher AUCs for the prediction of molecular diagnosis of AMR (AMR^Q>1^). Furthermore, besides the ptc score, only the AUC for the ptc^%^ and the new ptc^35%^ cut-off remained significantly associated with the prediction of molecular AMR, as follows: reference ptc^%^: AUC 0.76 (95% CI: 0.56–0.96, *p* = 0.04), ptc score: AUC 0.81 (95% CI: 0.63–0.98, *p* = 0.02), ptc^35%^: AUC 0.75 (95% CI: 0.54–0.96, *p* = 0.04), and ptc^50%^: AUC 0.69 (95% CI: 0.46–0.93, *p* = 0.13; Figure 4B, Table 2).

## 3. Discussion

This multicentric retrospective analysis was driven by our hypothesis that the current, empirically derived cut-off for diffuse ptc (50%) may not ideally reflect molecular AMR diagnosis and disease activity. Through comprehensive re-assessment of 63 allograft biopsies, we discovered that a ptc extent threshold of 35% correlates better with AMR-associated intragraft gene expression profiles compared to the established ptc^50%^ cut-off. The new ptc^35%^ cut-off resulted in higher AUCs for the prediction of significant AMR-associated gene expression in two independent cohorts, re-assessed by two experienced nephropathologists. If reproducible, our findings may lay ground for further studies to prospectively assess a new ptc cut-off and help to resolve current discrepancies between intragraft molecular gene expression platforms and light microscopy findings.

MVI is currently at the center of scientific attention, as presented by the Banff 2022 meeting report and the new AMR category definitions [9,15,16]. With the creation of the MVI+, DSA- category, the 2022 Banff meeting report underlined the important role of microvascular inflammation assessment to differentiate various spectrums of antibody-mediated rejection [16], particularly in the absence of donor-specific antibodies. In an extensive multicentric study, Sablik et al. showed that DSA-, C4d- allografts with MVI lesions had a worse graft survival compared to patients with probable AMR, defined as DSA positivity and only mild MVI [17]. Considering the prognostic implications of these findings, comprehensive assessment of MVI lesions should be the aim. Therefore, in our study, we included both ptc extent as a semi-quantitative score and as a continuous parameter, facilitating a more granular ptc assessment and correlations with molecular rejection features. Notably, both the ptc score and ptc extent correlated better with AMR gene expression than the Banff g score and had only slightly weaker correlations than the combined MVI score.

Despite earlier recommendations, most available literature includes only the ptc score and not the ptc extent. In a previous study, we observed that diffuse ptc was an independent predictor of worse graft survival [8]. In line, we measured significantly higher AMR gene expression in allografts with diffuse ptc. Notably, the correlation between the ptc score and ptc extent was not congruent in all cases. In particular, in biopsies with a ptc score of 2, the ptc extent ranged from 25% to 60%. Furthermore, all biopsies with a ptc extent of >35% had a ptc score of at least 2. Similar gene expression levels were measured in patients with a ptc extent between 35% and 50% compared to those with ptc ≥ 50%—supporting our hypothesis that the former cut-off may exclude some patients with substantial AMR gene expression.

The interpretation of inflammation in the peritubular capillary network can be a challenging task. Although traditionally considered a feature of AMR, ptc can be found in other types of allograft injuries, including TCMR and BKPyVAN [5,6,7,18,19]. In a previous study [8], we assessed that 48% of TCMR biopsies had ptc scores > 0, and as recently summarized by Varol et al. [5], even higher scores (≥2) can be observed in up to 25% of cases. In our study, which focused on AMR biopsies, we confirmed a strong association between the percentage of involved peritubular capillaries and intragraft AMR-associated gene expression. Proving this concept, we measured the highest AMR gene expression in biopsies re-assessed as AMR. Yet, reflecting the limited specificity, similarly high AMR gene expression was also observed in biopsies with mixed rejections.

Transplant glomerulopathy is considered a hallmark histological feature of chronic AMR and a known indicator of reduced graft survival [20,21,22]. To address the question if cg influences our new suggested ptc threshold, we performed a subgroup analysis of all biopsies with a cg score > 0. The higher AUCs with the ptc^35%^ threshold were reproducible in this subgroup, indicating that our main findings are also applicable to patients with chronic AMR.

The presented gene sets in our exploratory group are based on the 2017 Banff recommendations and represent precursors of the currently commercially available B-HOT panel [23]. Compared to other studies with a 770-gene set [12,13], we included a 208-gene involving panel for the exploratory cohort, suggesting further clinical studies with smaller and potentially more cost-effective gene sets.

There are two important strengths of our study. First, we comprehensively assessed different extents of peritubular capillaritis from biopsies re-evaluated by two experienced nephropathologists and combined conventional histology with intragraft gene expression analysis performed with commercially available diagnostic platforms. Secondly, we validated our findings by including an independent, external cohort. The correlations between light microscopy findings and gene expression were similar in both cohorts, confirming the reproducibility when using this diagnostic platform and the selected gene sets.

Nevertheless, our study is limited by sample size. Meaningful correlations with clinical endpoints were not possible; therefore, we cannot predict whether diffuse ptc according to our newly suggested cut-off results in worse graft survival. In addition, we only included indication biopsies in the exploratory cohort and half of the biopsies were follow-up biopsies. We therefore cannot rule out that AMR treatment might have affected gene expression profiles in some patients. Yet, before the initiation of anti-CD38 regimens, effective treatments, especially for late AMR, were limited [24,25]. Our study included selected allograft biopsy cases, possibly not representing the full spectrum of biopsies in a clinical setting. However, assessment of distinct cases is necessary to gain insights into pathophysiological processes. Furthermore, we were not able to reproduce the new ptc^35%^ cut-off in the recently proposed DSA-, CVD- MVI+ group because the majority of patients had DSAs at the time of the biopsy.

In conclusion, a new ptc cut-off ≥ 35% reflected molecular phenotypes of AMR more accurately than the ptc 50% cut-off in two representative patient cohorts from European transplant centers. We suggest that future studies with larger cohorts should prospectively investigate the impact of the ptc extent, and the new ptc^35%^ cut-off on clinical endpoints, including graft survival.

## 4. Materials and Methods

### 4.1. Patients and Inclusion Criteria

For this multicentric retrospective study, an exploratory cohort of 19 patients, with local biopsies performed because of suspected AMR (indication biopsies for allograft dysfunction and/or proteinuria) and follow-up (diagnostic) biopsies, corresponding to 38 samples, was analyzed (Medical University of Vienna and Ordensklinikum Linz, Linz, Austria). Part of the study cohort was initially screened for a multicenter trial focusing on novel transplant glomerulopathy classifications using digital pathology [14]. Inclusion criteria for the current study were as follows: (i) local biopsy because of clinically suspected AMR, (ii) adequate biopsy specimen for re-quantification of peritubular capillaritis, and (iii) sufficient archived material for molecular studies. We collected clinical data on the following parameters: date of transplantation, date of kidney biopsy, serum creatinine, and donor-specific antibody (DSA) status at transplantation and biopsy (Single Antigen Bead or Flow-PRA method) [26]. As an external validation cohort, we included 25 biopsies diagnosed with AMR from a partner institution, Erasmus MC, Rotterdam, The Netherlands. Due to the exploratory nature of the study and limited available samples with gene expression profiles, no sample size calculation was performed.

### 4.2. Exploratory Cohort and Intragraft Gene Expression

From 38 biopsies, ptc was assessable and sufficient material for gene expression analysis was available. All biopsies were digitally scanned and re-evaluated for Banff rejection diagnoses and single lesion scores by an experienced nephropathologist (M.M.), blinded to the clinical data. A partner institution (University of Alberta, Department of Laboratory Medicine and Pathology) in Edmonton, Canada, performed gene expression analysis of the exploratory cohort. The methodology is described in full detail elsewhere [12,13]. In short, three RNAse-free sections of 20 microns were de-paraffinized with subsequent RNA extraction using the RNeasy FFPE Kit (Qiagen, Hilden, Germany). RNA quantity was assessed with the NanoDrop 2000c spectrophotometer (NanoDrop Technologies, Wilmington, DE, USA). Molecular AMR diagnosis was defined based on previously published gene sets [10]. In total, 208 (exploratory group) and 784 (validation cohort) different genes were analyzed. All included genes and genes selected for aggregate AMR scores are listed in Supplemental Tables S1 and S2. Gene set expression was quantified using the nCounter XT Formulation with the NanoString nCounter FLEX Dx Analysis System as per manufacturer recommendations (with input RNA of about 100 ng) [12]. Raw gene expression counts were quality controlled and normalized using the nSolver Analysis Software version 4.0 (NanoString Technologies, Seattle, WA, USA) and manufacturer’s included positive and negative controls. Data were first normalized to the positive controls, followed by normalization to four housekeeping genes.

### 4.3. Validation Cohort and Intragraft Gene Expression

The validation cohort consisted of 25 AMR biopsies originating from the Erasmus MC, Rotterdam, The Netherlands, and was a part of a previously described cohort [27,28]. Gene expression analysis of the validation cohort was performed at the Department of Pathology in Rotterdam, as previously described [27,28]. In this group, the B-HOT panel encompassing 784 genes was used. To compare both cohorts, original gene expression results from the validation cohort were normalized to results from the exploratory cohort. Biopsy images from the validation cohort were transferred digitally, and scored as well as ptc extent were re-evaluated by a second experienced nephropathologist (N.K.), blinded to the clinical results.

### 4.4. Statistics

Discrete data were presented as counts and percentages. Continuous data were given as mean and standard deviation, or median and IQR, respectively. For univariate analyses, we applied Fisher’s exact, chi-square, Mann–Whitney U, and *t*-tests, as appropriate. Correlations were analyzed with the Spearman correlation coefficient. We calculated the geometric mean of the normalized counts to quantify aggregate gene score expression for each biopsy. AMR gene set expressions were compared between different histological diagnoses and ptc categories. To test the correlation of gene expression levels and histological scores, AMR gene expression levels of all biopsies were analyzed as a continuous parameter. AMR gene scores above the first quartile (AMR^Q>1^) were considered as positive values for ROC analysis. For statistical analysis, we used IBM SPSS Statistics (version 25, SPSS Inc., Chicago, IL, USA) and GraphPad Prism (version 8.40, GraphPad Software, La Jolla, CA, USA). A two-sided *p*-value < 0.05 was considered statistically significant. Different receiver operating characteristic curve were compared with MedCalc online (https://www.medcalc.org/en/calc/comparison_of_independentROCtest.php, accessed on 6 October 2025) [29], based on [30,31].

### 4.5. Ethics Committee

The study was conducted in accordance with the Declaration of Helsinki and approved by the Ethics Committee of the Medical University of Vienna (protocol code: 1572/2020, date of approval: 1 July 2020) for studies involving humans (https://www.meduniwien.ac.at/web/ueber-uns/ethikkommission/, accessed on 6 October 2025). Underlying clinical and gene expression data can be made available only upon reasonable request.

## Figures and Tables

**Table 1:** Description of included biopsies (exploratory cohort).

Parameters	Biopsies (N = 38)
Findings at transplantation *	
Recipient age at transplantation, mean ± SD	46.6 ± 16.0
DSAs at transplantation, N (%)	2 (5.3%)
Diagnosis	
No rejection/normal biopsy, N (%)	4 (10.5)
AMR, N (%)	26 (68.4)
Mixed rejection, N (%)	5 (13.2)
TCMR, N (%)	3 (7.9)
Findings at the time of biopsy	
DSAs detectable at biopsy, N (%)	24 (66.7)
Months after Tx, median (IQR)	4.2 (1.1–42.3)
Serum creatinine at 1st biopsy, mg/dL, median (IQR) *	2.2 (1.9–2.8)
Serum creatinine at 2nd biopsy, mg/dL, median (IQR) *	2.6 (2.1–4.7)
Banff lesion scores	
Peritubular capillaritis (ptc) extent (%)	25 (0–40)
Peritubular capillaritis (ptc) score, median (IQR)	1 (0–2)
Glomerulitis (g), median (IQR)	2 (1–2.5)
MVI score (g + ptc), median (IQR)	4 (1–4)
Chronic glomerulopathy (cg), median (IQR)	1 (0–3)
Interstitial inflammation (i), median (IQR)	0 (0–1)
Total inflammation (ti), median (IQR)	1 (1–2)
Tubulitis (t), median (IQR)	0 (0–1)
Intimal arteritis (v), median (IQR)	1 (0–2)
Interstitial fibrosis (ci), median (IQR)	1 (1–2)
Tubular atrophy (ct), median (IQR)	1 (1–2)
Vascular fibrous intimal thickening (cv), median (IQR)	0 (0–1)
Arteriolar hyalinosis (ah), median (IQR)	0 (0–0)

* corresponding to 19 included renal transplant recipients. Abbreviations: Ah: arteriolar hyalinosis, AMR: antibody-mediated rejection, cg: chronic glomerulopathy, ci: interstitial fibrosis, ct: tubular atrophy, cv: vascular fibrous intimal thickening, DSA: donor-specific antibodies, g: glomerulitis, i: interstitial inflammation, IQR: interquartile range, MVI: microvascular inflammation (g + ptc), N: number, ptc: peritubular capillaritis, TCMR: T-cell-mediated rejection, t: tubulitis, ti: total inflammation, Tx: transplantation, and v: intimal arteritis.

**Table 2:** ROC findings for the prediction of AMR^Q>1^ compared between both cohorts.

Variable	Prediction of AMR^Q>1^ (AUC, 95% CI)	
	Exploratory Cohort	Validation Cohort
PTC score	0.81, CI: 0.67–0.93, *p* = 0.004	0.81, CI: 0.63–0.98, *p* = 0.02
PTC extent (%)	0.83, CI: 0.70–0.96, *p* = 0.002	0.76, CI: 0.56–0.96, *p* = 0.04
PTC extent (50%)	0.74, CI: 0.56–0.90, *p* = 0.03	0.69, CI: 0.46–0.93, *p* = 0.13
PTC extent (35%)	0.78, CI: 0.63–0.93, *p* = 0.009	0.75, CI: 0.54–0.96, *p* = 0.04

Abbreviations: AMR: antibody-mediated rejection, AUC: area under the curve, CI: confidence interval, and PTC: peritubular capillaritis.

## Data Availability

The data presented in this study are available on request from the corresponding author. The data are not publicly available due to data being stored at the first and last author’s institution.

## Supplementary Materials

The following supporting information can be downloaded at: https://www.mdpi.com/article/10.3390/ijms262210945/s1.

## Abbreviations

The following abbreviations are used in this manuscript:

ah	Banff score arteriolar hyalinosis
AMR	antibody-mediated rejection
AMR^Q>1^	AMR-associated gene expression above the 1st quartile
AUC	area under the curve
cAMR	chronic antibody-mediated rejection
caAMR	chronic active antibody-mediated rejection
cg	Banff score chronic glomerulopathy
ci	Banff score interstitial fibrosis
CI	confidence interval
ct	Banff score tubular atrophy
cv	Banff score vascular fibrous intimal thickening
DSA	donor-specific antibodies
FFPE	formalin-fixed paraffin-embedded
g	Banff score glomerulitis
i	Banff score interstitial inflammation
MVI	microvascular inflammation
ptc	peritubular capillaritis
ptc^%^	ptc quantified as %
ptc^35%^	diffuse ptc extent cut-off defined as ≥35%
ptc^50%^	diffuse ptc extent cut-off defined as ≥50%
ROC	receiver operating characteristic
t	Banff score tubulitis
ti	Banff score total inflammation
TCMR	T-cell-mediated rejection
v	Banff score intimal arteritis
