# Supplementary material for: Molecular Diagnostics Supporting a ≥35% Diffuse Peritubular Capillaritis Extent Threshold for Diagnosis of AMR—A Retrospective Dual Center Study
Source: Int J Mol Sci. 2025 Nov 12;26(22):10945. doi: 10.3390/ijms262210945 (PMC12652923; doi:10.3390/ijms262210945)
Supplement: Supplementary file 1 [file ijms-26-10945-s001.zip › ijms-3943746-supplementary.pdf]

Supplements

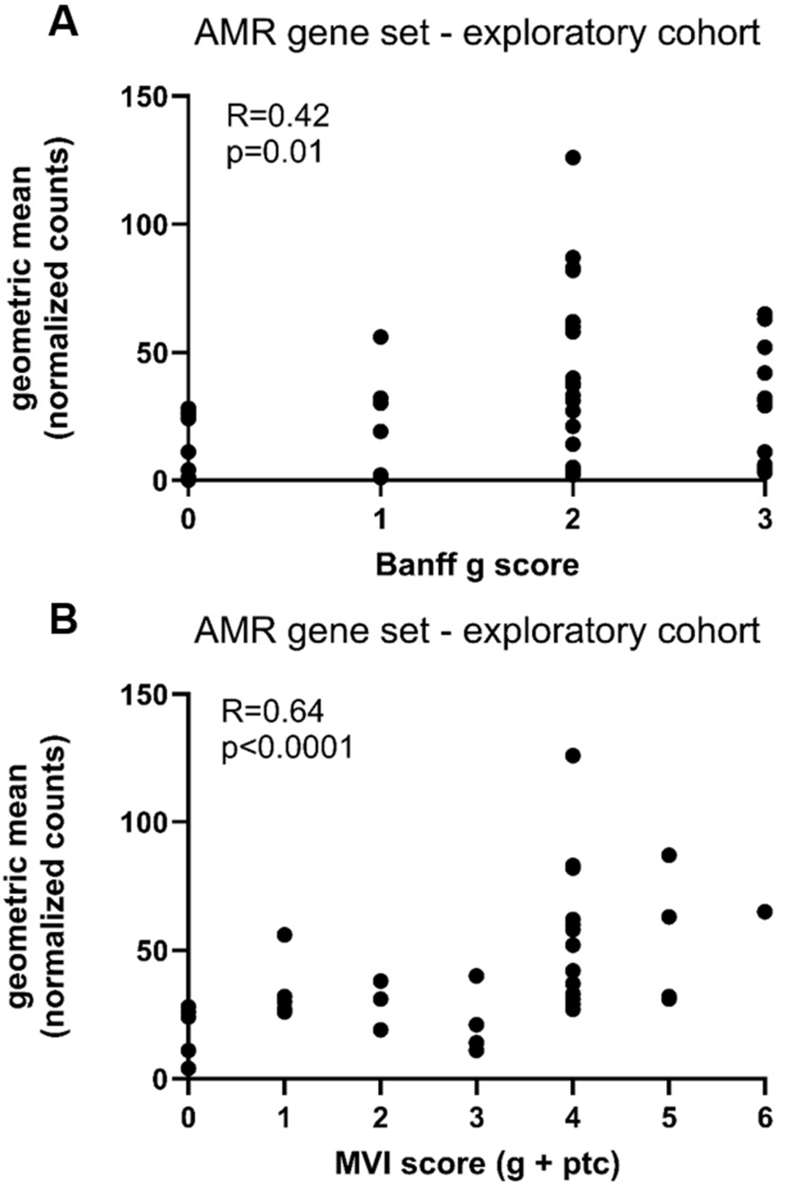

Figure S1. Correlation between AMR gene expression, Banff glomerulitis (g) score and the combined microvascular inflammation (MVI) score.

Figure S1A: Spearman correlation between intragraft AMR gene expression levels and the Banff glomerulitis (g) score in the exploratory cohort. Figure S1B: Spearman correlation between AMR gene expression and the combined MVI score (sum of Banff g and ptc score). Abbreviations: AMR: antibody-mediated rejection, g: glomerulitis (Banff score), MVI: microvascular inflammation (g+ptc) score, ptc: peritubular capillaritis (Banff lesion score).

Supplemental Table S1: All included single genes

Genes tested in the exploratory cohort (Vienna, Linz)	ACKR1, ADAMDEC1, AICDA, AIM2, AIRE, ANKRD22, AOAH, APOL1, APOL2, ARG1, ASB15, ATXN3, B3GAT1, BCL2, BCL2A1, BCL6, BKV, BTLA, CAV1, CCL21, CCL4, CCL5, CCR4, CCR5, CCR7, CD163, CD244, CD274,

	CD28, CD34, CD3D, CD4, CD68, CD72, CD74, CD84, CD86, CD8A, CD8B, CD96, CDH13, CDH5, CDKN2A, CHCHD10, CITED4, CLEC4C, COL1A1, COL3A1, COL4A1, COL4A3, COL4A4, COL4A5, COL5A1, CPA3, CTLA4, CTSS, CX3CR1, CXCL10, CXCL11, CXCL9, CXCR6, DUSP2, EDA, EEF1A1, EHD3, EOMES, EPO, EZH2, FAM26F, FCER1A, FCGR1B, FCGR3A, FGD2, FGFBP2, FJX1, FN1, FOXP3, GATA3, GBP1, GBP5, GEMIN7, GIMAP5, GNLY, GZMB, HAVCR1, HAVCR2, ICOS, IDO1, IFI30, IFNA1, IFNG, IGHA1, IGHG1, IGHG2, IGHG3, IGHG4, IGHM, IGKC, IGLC1, IKZF2, IL10, IL12RB1, IL17A, IL18BP, IL1RL1, IL2, IL21, IL21R, IL4, ITGAX, ITGB6, K15, KAAG1, KLF4, KLHL13, KLRF1, LAG3, LAIR1, LAP3, LCN2, LCP2, LGALS3, LTA, LTB, LTBR, LTF, MALL, MEGF11, MET, MIF, MRC1, MS4A1, MS4A4A, MS4A6A, MS4A7, MYB, MYBL1, NFATC1, NFKBIA, NFKBIZ, NNMT, NOS2, NPHS1, NPHS2, OSMR, PALMD, PDCD1, PDCD1LG2, PDPN, PECAM1, PHEX, PLA1A, PLAT, PRDM1, PSMB10, PSME2, PSTPIP1, PTPN7, RAB40C, RARRES1, RARRES3, RHOJ, RNF149, ROBO4, RPS6, RPS6KB1, RXRA, SELE, SERINC5, SH2D1A, SH2D1B, SHROOM3, SIRPG, SLA, SLAMF8, SLC19A3, SLC22A2, SLC25A15, SLC4A1, SLPI, SOD2, SOX7, SP140, SPRY4, ST5, ST8SIA4, TAP1, TBX21, TEK, TGFB1, TGIF1, THBD, TIGIT, TMEM178A, TNF, TNFAIP3, TNFRSF18, TNFSF8, TPSAB1, TRAF4, TRIB1, UL83, VCAN, VEGFA, VMP1, VWF, WARS, WNT9A
Genes tested in the confirmation cohort (Rotterdam)	AICDA, MYD88, CD28, SMAD2, PIK3CD, ICOS, IGFL1, PDCD1LG2, HLA-DQB1, CD40LG, ZAP70, CTSW, CARD16, NNMT, LTF, BMPR1B, XAF1, POU2AF1, S100A8, IRF6, RHOJ, LOX, TLR3, IL15, MMP12, MMRN2, NFKBIZ, IGHG2, IL17RA, FYN, IL17F, SLAMF7, EBI3, FASLG, IL2, BTG2, VMP1, LAMP1, GATA3, ENG, CCL5, WARS, BASP1, LAG3, IL2RG, FAS, BTLA, NLRC5, STAT6, IFI6, CD163, P2RX4, TRAF4, IL23A, NPPB, IL10, IL1B, MUC1, IFNGR1, COL13A1, MIF, TRDC, LCP2, CXCR5, IL2RA, IL4, ANXA1, PTPN22, NKG7, GDF15, TGFBI, DCAF12, FCER1A, ABCC2, CD69, CARD8, CD45RB, IL22, NPPA, EVA1C, CFLAR, COL4A5, PHEX, MYC, TNFRSF1A, IGLC1, CD4, JAK1, FGFBP2, APOLD1, THEMIS, TGFB2, ERG, ERRFI1, FCER1G, KIR_Activating_Subgroup_1, TIMP1, TNF, FCAR, KLRD1, RAB40C, ITGAM, CD34, SIGLEC5, CD3G, RASSF9, RAPGEF5, CD81, KLHL13, PTPN6, GZMH, RUNX1, HYAL1, HEG1, PLAAT4, KLRG1, C1QA, ECSCR, ROBO4, TRDV3, IFIT1, ACVR1, SPRY4, TRAF6, PSMB8, LAIR1, BST2, FOXP3, IL27, S100B, TGFBR1, TM4SF18, IL1RAP, NFIL3, CD8A, HLA-DPB1, LGALS3, SH2D1B, CCR4, IL1A, SIGIRR, CTLA4, BCL6, IL5, IFNA1, AOAH, AREG, IL13, UMOD, BATF, CSF1, SLC11A1, HLA-DPA1, CALHM6, IL17RC, CD80, GZMB, CD86, SLC4A1, KAAG1, XCL1/2, TRAT1, BCL3, PPBP, GBP5, KRT19, IL7R, TRDN, ZEB1, CGAS, KDR, S100A9, ARG2, CXCL10, HSD11B1, TGFB1, FGD2, CD48, IMPDH1, LTB, RORC, LTA, BLK, CXCL5, DUSP2, KRT8,

	CD14, ANKRD22, MRC1, PECAM1, EPO, SPIB, IL1R1, BIRC3, FCGR3A/B, MIR155HG, MS4A6A, MASP1, BAX, SLAMF6, SERINC5, CD207, CHCHD10, TAPBP, APOE, EGFR, TNFRSF18, IKBKB, CTNNB1, IKBKG, MAPK12, CD58, FJX1, IL12RB2, IL18RAP, BMP6, SELL, TP53, RORA, CXCL11, GAPDH, CEACAM3, MAPK14, ISG15, ST8SIA4, CETP, IL17A, SP100, KLRB1, MAP3K1, TYK2, CD3D, BMP7, KLRK1, PDGFRB, SP140, IER5, PNOC, NOS2, JAK2, BRWD1, GIMAP5, TEK, CCR6, HLA-DRB1, LILRB1, CD19, NOS3, TLR5, LYVE1, HSP90AA1, RGN, IMPDH2, IFI44, PDGFA, AQP1, ABCB1, IL1RL1, CD247, ALOX15, CASP3, MICA, MT1A, SLC22A2, LY96, KLRC1, CLEC4C, NOTCH2, IL6R, XBP1, HNF1A, DEFB1, HDC, PTPRO, IFITM3, RAF1, VCAM1, TNFSF10, CHUK, LEF1, MS4A7, MAF, TNFRSF9, C5, KIR3DL1, HMGB1, SEMA7A, PALMD, CPA3, CD274, CD1D, LILRB4, TRIB1, MMP14, B2M, BMPR1A, S1PR1, CMV, KIT, CCL22, MET, TNFRSF1B, TFF3, HLA-DMA, C9, CMKLR1, FCRL2, MAPK8, HLA-E, IKZF1, MPIG6B, BMP2, FABP1, TAP1, MERTK, IL6, NFKB1, CD84, BDNF, ARRB2, SYK, MS4A2, HLA-DRA, HYAL2, HIF1A, LILRB2, CXCL16, FPR1, TCF7, SOX7, TMEM178A, TM4SF1, OASL, AXL, NFKB2, CX3CR1, CD7, BCL2A1, PSMB9, WNT9A, MALL, PPM1F, KIR_Inhibiting_Subgroup_1, CD8B, SDC1, RAG2, ALAS1, GNG11, CCL4, LDLR, IL1R2, CXCL8, BK_Ltag, SLPI, BCL2L11, MT2A, NLRP3, TLR4, CD82, IL10RA, ADORA2A, RELB, ISG20, CTSS, MX2, PIN1, COL4A3, ABCE1, NPDC1, MICB, EBV, CD27, PLK2, IL27RA, JUN, TNFRSF14, MAPK3, STAT3, EMP3, BMP4, VEGFA, IL21, IFNG, ASB15, ICAM2, FADD, MEF2C, SERPINA3, LRRC32, TIGIT, MYB, PPP3CA, NFATC2, CXCL1/2, DNMT3A, TLR2, SAMHD1, PSMB10, RPS6KB1, F3, ACTA2, SCGB1A1, PTGS2, SELPLG, NFAM1, AQP2, CASP8, GBP2, CCL18, PSME1, CXCL9, TREM1, TANK, CRHBP, SOCS3, IGHA1, ADAM8, LST1, IRF4, CITED4, FKBP1A, GNLY, LCN2, TNFSF9, VCAN, APOL2, CCR5, TNFSF4, BCL2L1, PAX5, TGFBR2, INHBC, AGT, RAMP3, MS4A4A, IDO1, MEGF11, CD45R0, COL3A1, RXRA, CD24, KLF2, SFTPB, LTBR, STAT4, ATF3, DDX50, PSTPIP1, ABCA1, IFI27, SLC12A3, NFATC1, BMPER, CD70, IL23R, CFH, HPRT1, CSF3R, NFKBIA, CH25H, ACVRL1, NCAM1, IL2RB, CAV1, EZH2, HLA-A, AGER, MCM6, NOX4, TLR8, CRP, CCL3/L1, IL18BP, GZMK, STAT5A, PF4, IKZF2, MAPK13, JAK3, ITGB2, PDPN, B3GAT1, CD209, CD40, SFTPD, SOCS1, IL12RB1, CXCL2, KLRF1, SFTPA2, CIITA, PROX1, CD38, VWF, RNF149, CXCL14, IFI30, NPHS2, CDKN1A, COL4A4, SMARCA4, SLC19A3, NOTCH1, IL1RN, ARG1, CCL13, EDA, BLNK, TPMT, FOSL1, EGR1, SERPINE1, CD5, TLR9, RPL19, TIPARP, MX1, TOX2, FOXO1, PADI4, CD83, MASP2, TNFSF8, CXCR6, BTK, MYOM2, ST5, HLA-DQA1, CCR7, CD2, IFNAR2, COL4A1, IRS1, PTGER4, FLT3, CD59, CASP1, SKI, PSME2, AIRE, HFE, SMAD5, IL17RB, AHR, MEOX1, RELA, MYBL1, TNFAIP6, TBX21, CCR1, CD96, TNFRSF4, FCGR2B, IGHG1, CSF2RB, HAVCR1,

	EPAS1, TNFRSF17, CDH13, TNFSF14, IL16, IRF1, IFITM1, MAPK11, CD6, SLA, AGR2, TFRC, CCL20, PSEN1, CXCL12, INPP5D, KIR3DL2, SMAD3, SELP, CRIP2, LAP3, CD3E, CCR2, IGHG3, TNFSF18, IGF1, AIM2, DNMT1, CCL21, HLA-G, ADAMDEC1, SRC, IL18, FCGR1A, TRIM22, THBS1, IL21R, MCAM, PRDM1, IRF8, PRF1, MTOR, THBD, TNFAIP3, ALOX5, IL6ST, PTPRC, HLA-C, CFI, SHROOM3, GBP4, AKR1C3, HSPA12B, LAYN, APOL1, HDAC6, PTPN2, CXCR4, PTX3, MME, BCL2, CD55, CD72, PLA1A, LRP2, LCK, MMP9, CD276, EOMES, SMAD4, KIR_Inhibiting_Subgroup_2, CCL2, PLAUR, ICOSLG, CX3CL1, ITGA4, CCR3, SFTPC, HDAC3, ICAM1, NR4A1, TNC, CD22, PTPN7, PDCD1, GZMA, SERPING1, KLF4, KITLG, IL7, C5AR1, TGIF1, SLC25A15, RARRES1, HLA-B, S100A12, TBK1, GBP1, SLAMF8, C3AR1, CD46, ITGAX, CD244, C1QB, IRF7, FAM30A, NOD2, TAP2, CASP4, EEF1A1, HLA-DRB3, CTSL, HLA-F, CFB, IL10RB, IFITM2, CCL19, IGKC, BATF3, GEMIN7, IGF2R, CD47, EHD3, MARCH8., ITGB6, IL12B, TPSAB1/B2, OSMR, TCL1A, NCR1, REL, RTN4, PLAT, FCGR2A, ATXN3, HLA-DMB, RHOU, SH2D1A, CXCR3, MS4A1, PLAU, AGR3, CCR10, SIRPG, CDH5, C1S, VEGFC, ACKR1, MBP, ANKRD1, HAVCR2, NOD1, LHX6, CXCL13, CSF2, IGF1R, IL4R, IGHG4, IGHM, KIR_Activating_Subgroup_2, STAT1, ADGRL4, MYL9, ALDH3A2, CD160, CR1, ARHGDIB, ADAMTS1, PVR, FN1, IL33, OR2I1P, RASIP1, BK, LIF, CD74, SERTAD1, FOS, SOST, RGS5, CSF3, IFNGR2, IFNAR1, ATM, HK2, CD45RA, RPS6, CD79A, SELE, CD68, COL1A1, CD44, STAT5B, TLR7, SOD2, PIK3CG, C3, CCL15, NPHS1, IGFBP1, VSIR, IL12A, POS_C(8), POS_A(128), POS_F(0.125), POS_D(2), POS_B(32), POS_E(0.5), NEG_C(0), NEG_D(0), NEG_E(0), NEG_A(0), NEG_H(0), NEG_G(0), NEG_F(0), NEG_B(0), G6PD, OAZ1, ABCF1, TBP, POLR2A, NRDE2, GUSB, TBC1D10B, SDHA, UBB, PPIA, STK11IP,

Supplemental Table S2: Singe genes included in the AMR gene sets

Genes used for the AMR gene set in the both cohorts	ACKR1, CAV1, CD34, CD74, CDH13, CDH5, CX3CR1, CXCL11, FGFBP2, GATA3, GNLY, IFNG, KLF4, KLRF1, MALL, MYBL1, PALMD, PECAM1, PLA1A, PLAT, PSMB10, RHOJ, ROBO4, RPS6, RPS6KB1, SELE, SH2D1B, SOX7, TBX21, TEK, THBD, TNF, TRIB1, VWF

Abbreviations: AMR: antibody-mediated rejection

Supplemental Table S3: Correlation of AMR gene expression and Banff single lesions

Banff lesion score	Correlation with intragraft AMR gene expression
g_score	R=0.42, p=0.01
cg_score	R=0.18, p=0.27
i_score	R=0.19, p=0.26
ci_score	R=0.11, p=0.51

ti_score	R=0.15, p=0.39
ct_score	R=0.15, p=0.38
t_score	R=0.06, p=0.75
cv_score	R=0.02, p=0.90
ah_score	R=-0.03, p=0.88
v_score	R=0.24, p=0.17

Abbreviations: ah: arteriolar hyalinosis, AMR: antibody-mediated rejection, cg: transplant glomerulopathy, ci: interstitial fibrosis, ct: tubular atrophy, cv: vascular fibrous intimal thickening, g: glomerulitis, i: interstitial inflammation, t: tubulitis, ti: total inflammation, v: intimal arteritis
